# Sclerostin and Wnt Signaling in Idiopathic Juvenile Osteoporosis Using High-Resolution Confocal Microscopy for Three-Dimensional Analyses

**DOI:** 10.3390/children11070820

**Published:** 2024-07-04

**Authors:** Renata C. Pereira, Kathleen J. Noche, Barbara Gales, Zhangying Chen, Isidro B. Salusky, Lauren V. Albrecht

**Affiliations:** 1 Department of Pediatrics, David School of Medicine, University of California Los Angeles, Los Angeles, CA 90024, USA; rpereira@mednet.ucla.edu (R.C.P.); knoche@mednet.ucla.edu (K.J.N.); bgales@mednet.ucla.edu (B.G.); isalusky@mednet.ucla.edu (I.B.S.); 2 Department of Pharmaceutical Sciences, School of Pharmacy and Pharmaceutical Sciences, University of California Irvine, Irvine, CA 92697, USA; zhangyc3@uci.edu; 3 Department of Developmental and Cell Biology, School of Biological Sciences, University of California Irvine, Irvine, CA 92697, USA

**Keywords:** Wnt signaling, osteoporosis, children, sclerostin, bone biopsy, immunofluorescence

## Abstract

Background: Idiopathic juvenile osteoporosis (IJO) is a rare condition characterized by low bone mass that can increase the risk of fractures in children. Treatment options for these patients are limited as the molecular mechanisms of disease initiation and progression are incompletely understood. Sclerostin inhibits canonical Wnt signaling, which is important for the bone formation activity of osteoblasts, and elevated sclerostin has been implicated in adult osteoporosis. Objective: To evaluate the role of sclerostin in IJO, high-resolution confocal microscopy analyses were performed on bone biopsies collected from 13 pediatric patients. Methods: Bone biopsies were stained with sclerostin, and β-catenin antibodies showed elevated expression across osteocytes and increased sclerostin-positive osteocytes in 8 of the 13 total IJO patients (62%). Results: Skeletal sclerostin was associated with static and dynamic histomorphometric parameters. Further, colocalization analyses showed that bone sclerostin colocalized with phosphorylated β-catenin, a hallmark of Wnt signaling that indicates Wnt inhibition. In contrast, sclerostin-positive osteocytes were not colocalized with an “active” unphosphorylated form of β-catenin. Conclusions: These results support a model that altered levels of sclerostin and Wnt signaling activity occur in IJO patients.

## 1. Introduction

Osteoporosis is a metabolic disease defined by deteriorating bone architecture that can lead to fractures [1,2]. This disease impacts millions worldwide and an estimated 10 million people in the U.S. alone [3]. Genetic diseases can lead to osteoporosis in children such as osteogenesis imperfecta, Bruck syndrome, Marfan syndrome, osteoporosis pseudoglioma syndrome, Ehlers–Danlos syndrome, and homocysteinase [4]. Osteogenesis imperfecta is the most common cause of primary osteoporosis in children; in contrast, idiopathic juvenile osteoporosis (IJO) is a rare form of osteoporosis that is characterized by recurrent fractures, bone deformities, and/or back pain [5]. Over the last few years, different molecular pathways that regulate bone turnover RANKL, Wnt signaling, and TGF-β have been identified as potential causes of juvenile osteoporosis [2,4,6,7]. Osteoporosis in IJO patients manifests early in life, between ages 2 and 14, and leads to bone pain and spontaneous fractures in the absence of trauma [6,8] and in the absence of family history of childhood bone disease [4]. The genetic mutations have not been identified and, as a result, animal models of IJO have not been developed, which limits progress to improve therapeutic interventions.

Work from our group and others defined key characteristics of IJO bone disease as low bone volume in addition to impaired trabecular architecture and turnover [5,9,10]. Interestingly, the low bone formation rates in IJO patients were independent of mineralization defects [8]. Both dual energy X-ray absorptiometry [11] and quantitative computed tomography (QCT) have enabled assessment of bone size and density across trabecular and cortical bone [8]. Despite this progress, the pathophysiology and mechanisms underlying IJO remain poorly defined and anti-resorptive agents like bisphosphonates may have long-term consequences [2,6,7,12,13].

Osteocytes secrete different factors that regulate bone turnover and mineral metabolism [7]. For instance, sclerostin is known to decrease bone formation [14,15] and contribute to adult osteoporosis [16,17]. Several therapeutics have emerged for the treatment of post-menopausal osteoporosis that target sclerostin [18,19,20,21]. Originally, the sclerostin encoding gene (SOST^−/−^) was discovered by loss-of-function mutations in two autosomal recessive disorders, sclerosteosis and van Buchem disease (VBD) [22,23,24], which are characterized by high bone mass and a thickening of cortical bone due to hyperactive osteoblasts [16,17]. SOST^−/−^ knockout mice recapitulate human bone disease as demonstrated by the high bone mass and increased bone mineral density, volume, and formation [25]. Upon secretion, sclerostin inhibits osteoblasts through the inhibition of canonical Wnt signaling [26,27]. Loss-of-function mutations in Wnt pathway proteins, Wnt ligands, or lipoprotein receptor protein 5 (LRP5) decrease bone formation and lead to bone fragility [26,27,28].

Sclerostin localization and expression in osteocytes of adult bone have primarily applied immunohistochemistry (IHC), which lacks the capabilities to perform colocalization analyses [29,30,31]. Despite extensive clinical analyses [18,19,20,21], central gaps remain in our understanding of sclerostin signaling from osteocytes in human bone tissues. The aim of the present study sought to extend our previous analyses of IJO patients [8] by investigating sclerostin and its impact on Wnt signaling activity. Herein, we applied immunofluorescence microscopy analyses of sclerostin and Wnt signaling activity markers in human IJO bone.

## 2. Materials and Methods

In this study, bone biopsies were evaluated across 13 IJO patients, including the 8 patients from our previous study [8]. IJO was diagnosed based on the criteria defined by ISCD [32], which uses bone mineral density (BMD) values by DEXA or computed tomography, as previously shown [8]. Iliac crest bone biopsy was performed after double tetracycline labeling with modified Bordier trephine (0.5 cm diameter) at the UCLA center [8]. Genetic testing was performed in 4/13 patients and ruled out osteogenesis imperfecta [33]. This study was approved by the UCLA Institutional Review Board.

### 2.1. Bone Biochemistry and Histomorphometry

Bone tissues were prepared by ethanol fixation (70%), alcohol dehydration, and xylene clearance, and finally embedded in methyl methacrylate. Tetracycline labeling of bone specimens was assessed in unstained sections (10 μm). Toluidine blue staining was used to evaluate static histomorphometric parameters in undecalcified bone sections (5 μm). Trabecular bone was used to assess primary bone histomorphometric parameters with the Osteometric system (OsteoMetrics, Decatur, GA, USA) under 200× magnification. For measurements of mineralized bone, areas with dark blue staining to define regions and osteoid measurements were included as defined by pale blue seams (1.5 μm width). Derived indices were calculated according to standard formulae and abbreviations and nomenclature followed those from the American Society for Bone and Mineral Research. Histomorphometry analyses were performed at the Connie Frank UCLA Bone Laboratory, as previously described [8,34]. Controls were double-tetracycline-labeled iliac crest specimens and metabolite levels of 31 pediatric patients with normal kidney function undergoing elective orthopedic surgery, as previously reported [8]. Biochemical parameters were evaluated by UCLA nephrologists and endocrinologists and included serum levels of calcium, phosphorus, 25(OH) vitamin D, alkaline phosphatase, and intact parathyroid hormone (PTH). Serum calcium (Ca), phosphorus (P), creatinine, and albumin were measured using an Olympus AU5400 analyzer (Tokyo, Japan) and PTH (pg/mL) concentrations by the first-generation immunometric.

### 2.2. Bone Immunohistochemistry (IHC) and Quantification of Osteocytic Sclerostin

IHC staining and quantification of sclerostin was performed in all the bone sections. Bone IHC quantification was correlated with bone histomorphometric and biochemical parameters. Immunostaining of bone proteins was adapted from a previous report [34]. Briefly, bone tissue sections were de-plasticized in xylene and chloroform, rehydrated in graded alcohol solutions, and partially decalcified in 1% acetic acid. Endogenous peroxidase activity was quenched in 3% hydrogen peroxide/methanol solution. Non-specific binding was blocked in avidin/biotin solution with non-immune serum with 5% normal horse serum and 1% bovine serum albumin. Sections were incubated with affinity purified polyclonal goat anti-monoclonal anti-human sclerostin (1:500) overnight at 4 °C in a humidified chamber. Sections were then incubated with biotinylated antibodies towards goat, mouse, or rabbit; incubated for 30 min with StreptABC Complex/HRP kit followed by AEC subtract chromogen and counterstained with Mayer hematoxylin. Negative controls omitted primary antibodies. IHC was repeated on all specimens for staining reproducibility. Quantification of IHC was performed using the number of osteocytes that were positive for sclerostin staining divided by the T.Ar or B.Ar at 20× magnification. Bone samples from five adolescent control subjects were used for IHC and IF comparison.

### 2.3. Immunofluorescence Microscopy Analyses

Immunofluorescence (IF) analyses were performed to visualize sclerostin expression in osteocytes at high resolution and how sclerostin levels were related to Wnt signaling activity. For these analyses, primary antibodies included sclerostin and β-catenin, phosphorylated (phos) or unphosphorylated (active) forms (Cell Signaling; D13A1) were used, and secondary antibodies included AlexaFluor 568 (red) or AlexaFluor 488 (green). DAPI stained the nucleus [35]. Images were acquired on a Zeiss Imager confocal microscope LSM900 (Zeiss, Oberkochen, Germany) and Zen processing software (Version 3.10). Image J (Version 9, https://fiji.sc/, accessed on 1 July 2024) was used to normalize brightness and contrast across primary antibody channels. Images were taken at 10×, 20×, and 63× objectives. Analyses of sclerostin secretion from puncta was performed across total sclerostin-positive cells in set fields based on pixels/mm^2^, manually selected to avoid areas with tissue damage, as in previous work [34]. For these calculations, secreted sclerostin punctate was calculated based on the diameter of vesicular structures across a single z-plane with background fluorescence removed after fluorescent intensity was normalized.

The diameters of puncta were manually quantified across bone unit areas at the same magnification, and statistical analyses included standard two-sided t tests with type I error < 5% considered as statistically significant. Figures show representative images, and normalized fluorescence measurements are presented as the mean ± SEM.

### 2.4. Statistical Analysis

All statistical analyses were performed using SAS version 9.4 and all tests were two-sided. For puncta diameter quantification across bone unit areas, standard two-sided t tests with type I error < 5% considered as statistically significant and ordinary *p* values were reported. Immunofluorescence images in figures are representative images and normalized fluorescence measurements are presented as the mean ± SEM.

## 3. Results

### 3.1. Biochemical Characteristics and Parameters of Bone Histomorphometry

Table 1 contains the biochemical clinical characteristics and demographics of the 13 IJO patients. Of these patients, 10 were male and 3 were female. The ages of patients ranged from 3 to 18 years, with a median age of 11.96 years. Serum calcium and phosphate levels were within the normal ranges previously reported [8]; overall serum alkaline phosphatase levels were median level 242 IU/L (interquartile 96- and 385). Serum 250HD and PTH were within the normal range in the vast majority of patients. Fracture locations are indicated in Table 1 and patients 6 and 13 presented with bone pain, bone deformities, and an absence of fractures. Table 2 and Table 3 contain the static and dynamic bone histomorphometry parameters, respectively. Trabecular bone formation (BFR/BS) and mineralization apposition rates were diminished in most of the patients. Decreased connection between trabeculae plates and a reduction trabecular thickness was observed across the majority of bone tissues, which is consistent with bone fragility [5]. Quantitative computed tomography was performed in eight patients and mean cancellous bone density was −2.48 + 0.4 mg/cm^3^ and DEXA total body Z score was −1.65 + 0.5 g/cm^2^ in the remaining patients.

### 3.2. Sclerostin Bone Staining

**Figure 1:**
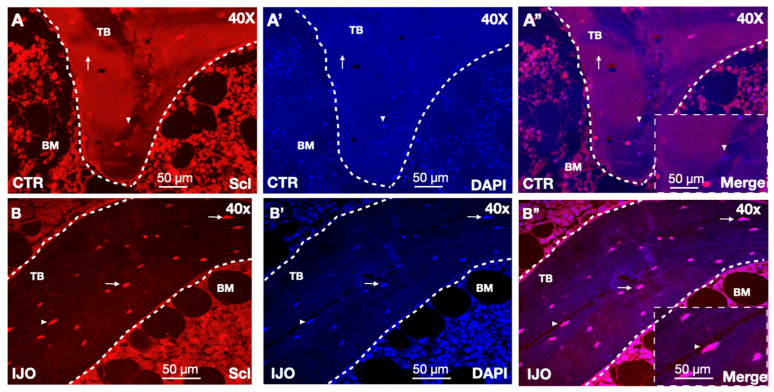
Sclerostin expressing osteocytes in IJO bone biopsies. (**A**) Staining with an anti-sclerostin antibody (red) was assessed in healthy control (CTR) bone at 40×. (**A’**) DAPI was used as a nuclear marker (blue). (**A”**) CTR bone staining of sclerostin and DAPI. (**B**) Staining with an anti-sclerostin antibody (red) was assessed in IJO. (**B’**) DAPI was used as a nuclear marker (blue). (**B”**) IJO bone staining of sclerostin and DAPI. Arrows and arrowheads (insets) denote corresponding cells in red and blue channels with osteocytes expressing high levels of sclerostin. Trabecular (TB) bone and bone marrow (BM) are indicated. Scale bars are shown as 50 µm.

To study the role of sclerostin in pediatric osteoporotic bone disease, we performed high-powered spinning disk confocal microscopy and immunofluorescence analyses of sclerostin stained biopsies from IJO bone (Figure 1). Sclerostin expression was increased in 8 of the 13 patients analyzed (62%) compared with healthy control bone as assessed by the number of sclerostin-positive osteocytes across bone unit areas (Figure 1A,B). Comparisons were made using the same total number of osteocytes in each biopsy (Table S1). These elevated levels of sclerostin staining were evident in bone imaging at low power and became even more striking under higher levels of magnification (Figure 1A,B). Together, these data demonstrate that IJO bone patients had increased sclerostin expression.

### 3.3. Sclerostin Secretion from Osteocyte Dendrite Vesicles

**Figure 2:**
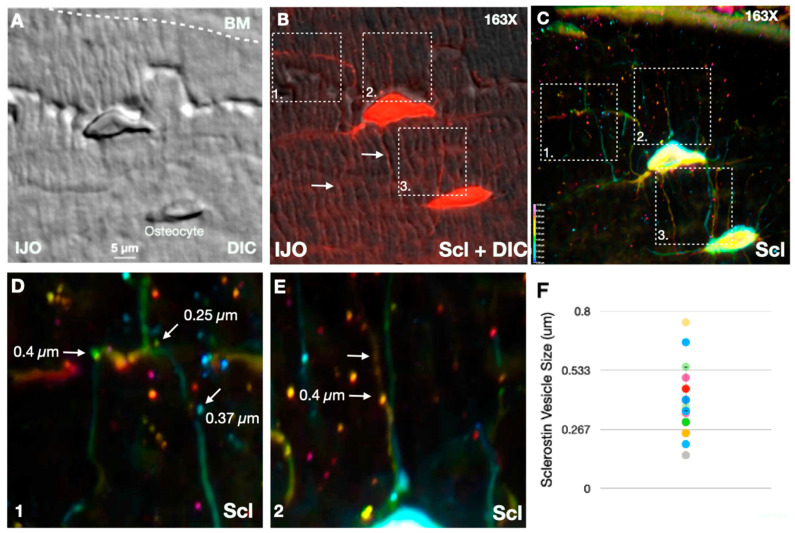
High resolution three-dimensional sclerostin staining in IJO osteocytes. (**A**) Phase contrast imaging (DIC). (**B**) DIC overlaid with immunofluorescence staining with sclerostin antibody (red) of bone biopsy. (**C**) Heatmap of pixel intensity for sclerostin fluorescence intensity across z-stacks in IJO osteocytes (163×). (**D**,**E**) Insights of heatmap immunofluorescence staining of sclerostin. Scale bars are shown as 5 µm and 1 µm. Colors represent z-stack slice. (**F**) Quantification of sclerostin vesicle sizes.

We previously reported that osteocytes were altered in monogenetic osteoporosis [10,31]. Thus, further analyses were performed at 163× magnification and sclerostin staining was analyzed using a three-dimensional heat map topology to enable the visualization within 10 µm in the z-plane. Osteocytes and associated dendrites were imaged using phase contrast to enable the visualization of dendrite morphologies throughout the bone matrix (Figure 2A). The overlay of phase contrast and sclerostin antibody staining demonstrates the expression of sclerostin within the cell body and along dendrite projections (Figure 2B and Figure S1). Interestingly, sclerostin dendrite staining was accompanied by discrete punctate structures that fell within the size prediction of secreted factors with a size of 0.4 µm. To visualize whether punctate sclerostin corresponded to individual dendrites, color coding was performed for each z-plane (Figure 2C). Sclerostin-positive punctate showed diameters between 0.25 and 0.40 µm and were found within proximity of a dendrite with the same color (Figure 2C,D), which could be regions of sclerostin secretion.

### 3.4. Sclerostin Associations with Biochemical Parameters of Bone Histomorphometry

Correlations between biochemical markers and bone histological variables showed that only serum alkaline phosphatase levels were associated with sclerostin staining in total bone areas (r = 0.65, *p* = 0.02). Bone volume (BV/TV) negatively correlated with sclerostin across bone areas (r = −0.62, *p* = 0.02) and tissue areas (r = −0.59, *p* = 0.04). Trabecular separation (Tb.Sp) was associated with sclerostin across the bone area (r = 0.58, *p* = 0.04). No correlations were observed with other static or formation bone parameters.

### 3.5. Sclerostin and Wnt Signaling

**Figure 3:**
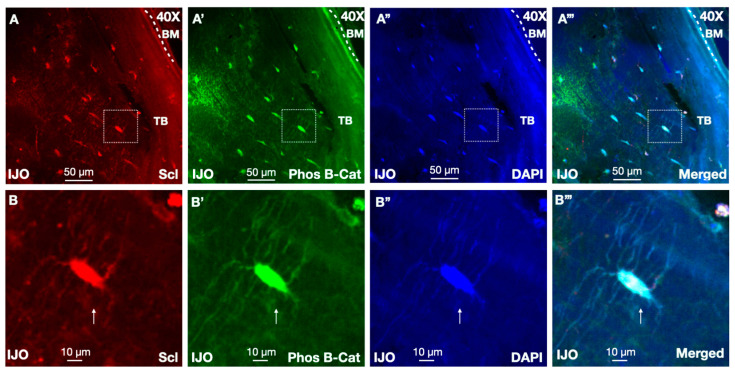
Sclerostin-positive osteocytes colocalize with phosphorylated β-catenin. (**A**) Bone biopsies stained with a sclerostin antibody (red), (**A’**) phosphorylated β-catenin (green), and (**A”**) DAPI (blue). (**A’’’**) displays merged channels. Arrows indicate corresponding cells where sclerostin-positive osteocytes and phos β-catenin staining. Dotted white boxes are insets that highlight colocalization from the corresponding image in the row below. Magnified regions of (**B**) sclerostin, (**B’**) phos β-catenin, and (**B”**) DAPI staining, in region marked from the row above. Trabecular (TB) bone and bone marrow (BM) are indicated. Scale bars are shown as 50 µm and 10 µm. (**B’’’**) Merge of panels (**B**–**B”**).

Osteocytes secrete factors to orchestrate bone remodeling and sclerostin is a known inhibitor of bone formation via Wnt signaling [27]. At the heart of the Wnt pathway is β-catenin, which is dynamically regulated by its post-translational modification state where β-catenin phosphorylation marks pathway inhibition. To specifically examine whether bone sclerostin, an inhibitor of Wnt signaling, had functional roles on molecular signaling, we performed colocalization analyses with β-catenin antibodies. Bone sections from patients with increased sclerostin expression by IHC were analyzed by IF staining with antibodies recognizing active β-catenin (unphosphorylated) or inhibited β-catenin (phosphorylated, phos) [36]. Analyses of phos β-catenin demonstrated high staining across osteocytes of patients with high levels of sclerostin expression, consistent with low Wnt signaling. Importantly, colocalization of sclerostin and phos β-catenin expression within osteocyte populations were high, which is consistent with a model whereby sclerostin inhibits Wnt canonical Wnt activity (Figure 3A,B).

**Figure 4:**
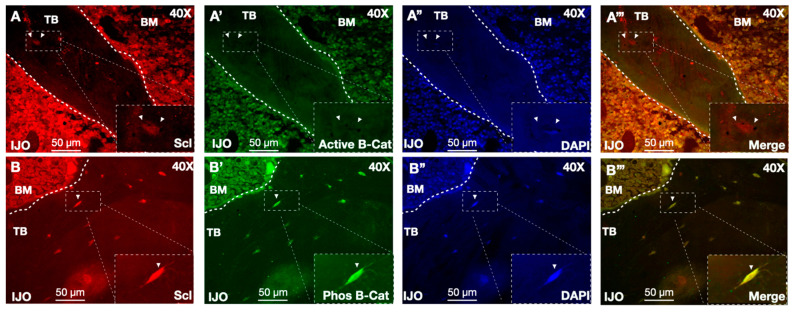
Sclerostin expressing osteocytes have low Wnt signaling activity. (**A**) IJO bone biopsies stained with a sclerostin antibody (red), (**A’**) active β-catenin (green), and (**A”**) DAPI (blue). (**A’’’**) displays the merged channels of (**A**–**A”**). Arrowheads indicate corresponding cells where sclerostin-positive osteocytes lack active β-catenin staining. (**B**) Bone biopsies stained with sclerostin, (**B’**) phosphorylated (phos) β-catenin (green), and (**B”**) DAPI (blue). (**B’’’**) displays merged channels of (**B**–**B”**). Arrows denote corresponding cells with colocalized sclerostin and phos β-catenin. Insets from dotted white boxes highlight colocalization. Trabecular (TB) bone and bone marrow (BM) are indicated. Scale bars are shown as 50 µm.

To further evaluate Wnt signaling, bone biopsies were stained with sclerostin and unphosphorylated β-catenin. Consistent with the model that sclerostin and Wnt activity were linked, sclerostin-positive osteocytes showed almost no staining with the unphosphorylated “active” β-catenin antibodies, further supporting that sclerostin inhibits Wnt signaling (Figure 4A,B). Altogether, these immunofluorescence datasets support that sclerostin and its downstream signaling pathway are altered in the present cohort of IJO patient biopsies.

## 4. Discussion

The present study harnesses the full value of bone biopsy through the application of high-powered immunofluorescence confocal microscopy for three-dimensional analyses. Sclerostin was the focus of our investigation as it has been implicated in adult osteoporosis. We demonstrated that increased staining of sclerostin was found in 8 of the 13 total IJO patients from the present small cohort (62%). Moreover, sclerostin expressing osteocytes also had higher levels of a marker for Wnt signaling inhibition. Together, these data highlight the use of three-dimensional imaging approaches for visualizing osteocyte secreted factors in bone biopsies and future investigations of sclerostin in IJO are warranted.

### 4.1. Sclerostin Secretion from Osteocytes

We showed that sclerostin proteins were loaded along dendrites and accumulated into distinct punctate structures prior to subsequent secretion. We deployed the use of high-resolution microcopy and phase contrast, which enabled the visualization of osteocyte structures throughout the bone matrix. Interestingly, dendritic processes formed fossils within the bone matrix. This was an interesting finding given the key roles of osteocytic dendrites in mechanical sensation [37,38] that could impact the functional rigidity of bone in IJO patients.

### 4.2. Bone Biopsies Provide a Rich Resource to Elucidate Mechanisms of Disease

Sclerostin contributes to adult osteoporosis, and sclerostin antibody-based therapies have been developed [18,19,20]. Despite this, there have been few investigations focused on the visualization of sclerostin in bone tissues. Indeed, studies in adult bone have relied only on immunohistochemistry [15,29,30,31]. In fact, sclerostin analyses by IF microscopy has been limited to mouse models [28]. This is due, in part, to technical challenges that have previously hindered the use of IF microscopy to image human bone. The present analyses provide new insight for the field studying pediatric osteoporosis using preserved bone biopsies that were stored in a −80 °C incubator. Future studies of sclerostin using IF staining in bone biopsies from differing stages of bone disease could lead to new molecular understanding to improve targeted precision medicine.

The characterization of IJO on a molecular level has been limited by a scarcity of bone biopsy samples in addition to the technical obstacles stated above. Given this, we applied IF microscopy to investigate the hypothesis that sclerostin may contribute to bone disease in children, as is previously reported in adults [16,17]. The present IF datasets reporting a marked elevation of sclerostin across trabecular and cortical bone areas provide new mechanistic insight into the pathophysiology of IJO (Figure 1) and suggests that similar mechanisms may play a role in adult and pediatric bone loss. Limitations of the current study are the small sample size and the lack of circulating sclerostin levels. Additionally, osteogenesis imperfecta and monogenetic osteoporosis could not be ruled out in the present analyses as the genetic screening may have missed these mutations in our patients.

Sclerostin inhibits bone formation through canonical Wnt signaling. The present analyses demonstrate that altered sclerostin levels in IJO coincided with Wnt signaling inhibition as marked by phos β-catenin fluorescence intensity. We report positive associations between bone sclerostin and serum levels of ALP, as has been previously reported with circulating sclerostin levels in studies of childhood obesity or in a hemodialysis cohort [39,40]. These data could support a model whereby the sclerostin-Wnt signaling axis may play a role in the reduced bone formation rates in IJO. However, it is important to note that osteocytic secretion of sclerostin is a biochemical signal response to mechanical stress. Thus, whether altered osteocyte mechanosensing contributes to sclerostin secretion in IJO remains to be an open question.

### 4.3. Future Applications of Imaging in Osteocyte-Secreted Factors of Native Environments

Previously our group has reported that patients with mutations in Wnt 1 have increased phos β-catenin levels in osteocytes when compared with mutations in PLS3 [31]. Similarly, elderly patients with low bone formation rates (~0.1) also have higher phos β-catenin levels. Other osteocyte-secreted factors have been reported to be elevated in monogenetic osteoporosis and renal osteodystrophy like FGF23 and DMP1 [31,41,42]. While DMP1 is found generally across all osteocyte populations, FGF23 expression is notably high in the periphery of the trabecular bone and sclerostin is found in deeper osteocyte populations [43]. Whether the location, differentiation state, or age of osteocytes regulates the secretion of key factors in disease remains to be an open field of research. However, these studies, together with the work shown here, highlight clear functions of osteocytes in bone health and disease. In summary, the present findings highlight the power of bone biopsy when combined with immunofluorescence confocal microscopy to elucidate new molecular mechanisms of bone disease.

## Figures and Tables

**Table 1:** Clinical characteristics and demographics of IJO patients.

	Sex	Age (yrs)	Ca (mg/dL)	PO4 (mg/dL)	ALP (IU/L)	PTH (pg/mL)	25OH (ng/dL)	Fracture Location	Fracture Number
**1**	M	6	9.9		189.0	13.0	55.0	tibia; wrist; femur	>5
**2**	M	14	9.7	4.8	188.0	36.0	36.0	ankle; wrist; femur; tibia; ulna; ribs	>10
**3**	M	8	9.3	5.4	234.0	20.0	54.0	compression; scoliosis	>3
**4**	M	14	9.8	5.6	141.0	40.0	39.0	compression fractures	>3
**5**	M	13	9.8	4.7	225.0	41.0	22.0	elbow; wrist; forearm; ankle; knee	>5
**6**	F	10	9.7	4.7	232.0	NA	NA	NA	
**7**	F	11	9.8	5.1	365.0	45.0	42.0	wrist; metacarpal; arm; scapula	>4
**8**	M	8	9.5	4.7	337.0	38.0	23.0	hip; vertebral; tibia	>3
**9**	M	13	10.2	4.8	385.0	33.0	30.0	fingers; arm	>3
**10**	M	15	10.2	4.6	280.0	25.0	40.0	vertebral; finger	>2
**11**	F	3	10.2	5.6	257.0	41.0	38.0	long bone; tibia; elbow	>3
**12**	M	13	10.0	4.1	228.0	27.0	33.0	wrist; thumb; vertebrae; compression	>6
**13**	M	18	9.9	4.1	96.0	22.0	46.0	NA	

**Table 2:** Markers of IJO patients and healthy control (C) range.

	BV/TV (%)	OV/BV (%)	OS/BS (%)	Ob.S/BS (%)	ES/BS (%)	Oc.S/BS (%)	Tb.Th (μm)	O.Th (μm)	Tb.Sp (μm)	Tb.N (mm)
**1**	13.6	0.4	4.9	1.1	5.2	0.2	96.0	3.4	609.6	1.4
**2**	18.1	1.1	7.7	2.2	13.3	0.9	104.9	7.2	473.9	1.7
**3**	23.6	1.6	13.2	0.6	0.8	0.3	132.8	8.0	429.5	1.8
**4**	31.2	2.2	10.6	1.3	13.8	1.9	77.6	8.2	171.6	4.0
**5**	20.5	0.9	8.4	0.9	0.0	0.0	131.2	7.2	508.2	1.6
**6**	26.8	2.1	19.3	5.3	3.6	1.0	144.7	7.8	395.3	1.9
**7**	12.6	2.0	12.0	3.9	0.6	0.0	104.3	8.6	721.6	1.2
**8**	16.9	1.6	18.5	0.5	1.6	0.1	89.8	3.9	442.4	1.9
**9**	23.5	4.2	31.6	2.6	3.8	1.2	122.9	8.1	399.9	1.9
**10**	26.7	4.1	31.0	8.6	7.0	1.8	141.4	9.3	388.2	1.9
**11**	16.3	1.3	10.4	2.5	0.4	0.0	81.1	5.0	418.2	2.0
**12**	31.0	1.2	9.6	3.9	1.6	0.5	129.2	8.1	288.0	2.4
**13**	28.0	2.9	14.3	1.3	2.1	1.0	117.3	12.0	302.2	2.4
**C**	8.9–34.3	0.2–5.8	4.3–37.0	-	0.5–4.3	-	91–177	2–13.2	351–737	1.1–2.2

Bone volume/tissue volume (BV/TV), osteoid volume/bone volume (OV/BV), osteoid surface/bone surface (OS/BS), osteoblast surface/bone surface (Ob.S/BS), eroded surface/bone (ES/BS), osteoclast surface/bone surface (Oc.S/BS), trabecular thickness (Tb.Th), osteoid thickness (O.Th), trabecular separation (Tb.Sp), and trabecular number (Tb.N).

**Table 3:** Bone markers of IJO patients and healthy control (C) range.

	MS/BS (%)	MS/OS (%)	MAR (μm/d)	Aj.AR (μm/d)	BFR/BS (μ^3^m/μ^2^m/y)	Mlt (Days)	Omt (Days)
**1**	3.7	75.3	0.8	0.6	10.1	6.1	4.6
**2**	3.0	39.3	0.8	0.3	8.9	22.7	8.9
**3**	5.2	39.4	0.9	0.3	16.5	23.3	9.2
**4**	4.6	43.3	0.9	0.4	15.5	20.5	8.9
**5**	5.4	64.2	1.0	0.6	19.6	11.3	7.2
**6**	5.2	26.7	0.5	0.1	9.4	58.3	15.6
**7**	3.4	28.3	0.7	0.2	8.3	45.4	12.8
**8**	7.5	40.4	0.7	0.3	19.5	13.6	5.5
**9**	18.3	58.1	0.8	0.4	50.3	18.5	10.7
**10**	17.8	57.6	1.0	0.6	67.6	15.6	9.0
**11**	2.0	19.2	0.7	0.1	5.4	35.2	6.8
**12**	3.4	35.2	0.9	0.3	10.5	27.2	9.6
**13**	5.0	34.6	0.6	0.2	10.1	62.3	21.6
**C**	2.2–19.0	7.3–66.2	1.1–1.5	0.14 ± 1.20	10–73.4	2.3–63.8	0.0

Mineralizing surface/bone surface (MS/BS), mineralizing surface/osteoid surface (MS/OS), mineral apposition rate (MAR), adjusted apposition rate (Aj.AR), bone formation rate/bone surface (BFR/BS), mineralization lag time (Mlt), and osteoid maturation time (Omt).

## Data Availability

The data presented in this study are publicly available and provided in Figures and Tables in the manuscript.

## Supplementary Materials

The following supporting information can be downloaded at: https://www.mdpi.com/article/10.3390/children11070820/s1, Figure S1: High resolution three-dimensional phase contrast Z-stacks with sclerostin staining in IJO; Table S1: Quantification of average sclerostin-positive osteocytes per patient; Video S1. Sclerostin staining with phase contrast in three-dimensional analyses within IJO bone biopsies; Video S2. Sclerostin staining by immunofluorescence with different z-planes pseudo colored.
