# Supplementary material for: Sclerostin and Wnt Signaling in Idiopathic Juvenile Osteoporosis Using High-Resolution Confocal Microscopy for Three-Dimensional Analyses
Source: Children (Basel). 2024 Jul 4;11(7):820. doi: 10.3390/children11070820 (PMC11276078; doi:10.3390/children11070820)
Supplement: Supplementary file 1 [file children-11-00820-s001.zip › children-3001664-supplementary.pdf]

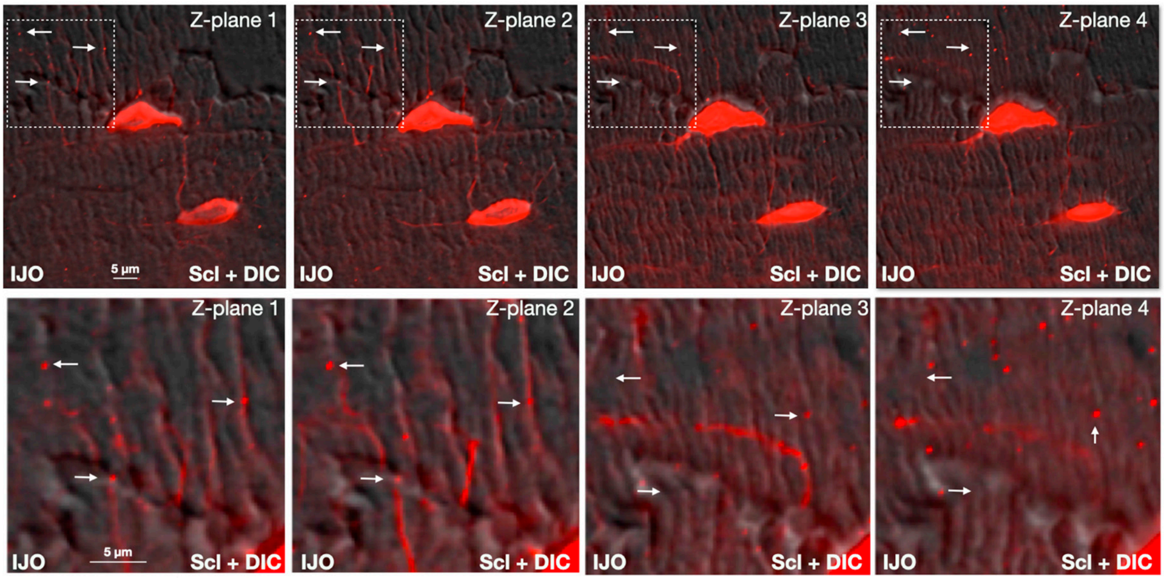


**Figure S1.** High resolution three-dimensional phase contrast Z-stacks with sclerostin staining in IJO. Phase contrast imaging (DIC) overlaid with immunofluorescence staining with sclerostin antibody (red) of bone biopsy across z-stacks, associated with Figure 2. Scale bars shown as 5 µm.

**Table S1.** Sclerostin-positive osteocytes across IJO patient bone. Quantification of IJO bone areas stained with sclerostin antibody and DAPI (nuclear). Values represent averages of cells across patient bone with over 100 cells per section.

| Patient | Scl-Positive/Total Osteocyte (%) |
|---|---|
| **1** | 42.1 |
| **2** | 7.63 |
| **3** | 14.4 |
| **4** | 36.2 |
| **5** | 11.9 |
| **6** | 29.1 |
| **7** | 76.0 |
| **8** | 67.0 |
| **9** | 49.4 |
| **10** | 100 |
| **11** | 56.2 |
| **12** | 51.1 |
| **13** | 52.6 |
